# Modifying the EPA’s New Power Plant Rules to Eliminate Unnecessary Reliability Risks

**DOI:** 10.1021/acs.est.3c04608

**Published:** 2023-07-21

**Authors:** David C. Rode, Jeffrey J. Anderson, Haibo Zhai, Paul S. Fischbeck

**Affiliations:** † Department of Social & Decision Sciences, Carnegie Mellon University, 5000 Forbes Avenue, Pittsburgh, Pennsylvania 15213, United States; ‡ Carnegie Mellon Electricity Industry Center, Carnegie Mellon University, 5000 Forbes Avenue, Pittsburgh, Pennsylvania 15213, United States; § RAND Corporation, 4570 Fifth Avenue, Suite 600, Pittsburgh, Pennsylvania 15213, United States; ∥ Department of Civil & Architectural Engineering, University of Wyoming, 1000 East University Avenue, Laramie, Wyoming 82071, United States; ⊥ Department of Engineering & Public Policy, Carnegie Mellon University, 5000 Forbes Avenue, Pittsburgh, Pennsylvania 15213, United States

**Keywords:** CO_2_ regulation, carbon capture and storage, net zero, resource adequacy, Section 45Q

When the Environmental Protection Agency (EPA) proposed new rules^1^ governing emissions from coal and natural gas power plants on May 11, 2023, it was the federal government’s third attempt (along with the Clean Power Plan and the Affordable Clean Energy rule) in the past decade to reduce carbon dioxide (CO_2_) emissions by requiring changes at existing coal and natural gas power plants. Historically, the United States has used nuclear power, coal, and natural gas—sources that can be operated on demand, without regard for whether the wind is blowing or the sun is shining—to generate reliable electricity. The 2030 decade, however, is expected to see a sizable portion of the nation’s aging nuclear power plants reach retirement age.^2^ Now, without careful modification, the EPA’s new proposal would push many existing coal and natural gas generators to retire as early as 2030, imperiling grid reliability.

Although we write primarily about the United States, we note a similar dynamic is playing out around the world. Countries such as Canada,^3^ the United Kingdom,^4,5^ Australia,^6^ and China^7−9^ have also taken recent steps to incentivize CO_2_ reduction beyond renewable energy using both market-based and regulatory approaches. These efforts are increasingly being made mindful of the impact of carbon-reduction mandates on electric-system reliability and in light of the fact that geopolitical issues in Ukraine have snarled energy supply chains in many parts of the world.

Renewable energy sources, such as wind and solar power, have become increasingly cost-effective on their own and emit no CO_2_, rightfully securing them a place at the table. Society, however, values reliability in electricity generation, and renewables—however clean and cost-effective—are simply not reliable without costly battery storage systems as backup,^10^ without employing clean hydrogen,^11^ or without greatly expanding the transmission grid.^12^ When people flip on their light switches, they expect the power to be there. How, then, are we to maintain reliability while reducing CO_2_?

Let us tackle one source of conflict up front: the reality is that the country’s coal power plants are aging out. Roughly one-third of U.S. coal-generating capacity has already retired over the past 20 years (for economic, environmental, and physical reasons), and the remaining coal power plants are, on average, nearly 45 years old.^2^ Even without any new regulation, a majority of these plants will have reached retirement age by the 2030–2040 time frame contemplated by the EPA’s proposed new rules.^13^ Europe, too, is working to reduce its reliance on coal-fired electricity. Simply exempting coal from further regulation is not a long-term solution. The prospects for nuclear power are also decidedly mixed. The production of nuclear power does not involve CO_2_ emissions, but the disposal of nuclear waste presents environmental issues that are not easily (or inexpensively) addressed. Perhaps more importantly, however, the track record for developing new nuclear power plants, given recent experience with projects in Finland (Olkiluoto 3) and in the United States (Vogtle 3 and 4), suggests that meeting climate targets beginning in 2030 via new nuclear generating capacity is a formidable undertaking. It is this intermediate period that presents the most challenges.

With nuclear and coal power plants retiring regardless of new EPA rule making, the burden of generating reliable, low-cost electricity falls increasingly on natural gas power plants. Fortunately, electricity produced by natural gas has nearly tripled since 2001. However, as with its previous efforts, the EPA’s latest attempt is a blunt instrument. While the proposed rules do not require that natural gas plants retire, the burdens they impose push plant owners in that direction, threatening grid reliability. The EPA may not be concerned, but organizations whose mandates explicitly include maintaining reliability have been vocal about efforts to undertake the nation’s transition to cleaner energy too quickly. The Federal Energy Regulatory Commission, the North American Electric Reliability Corporation, and the National Rural Electric Cooperative Association have each issued warnings about reliability in the wake of the EPA’s proposed rule.^14^

The EPA’s proposed new rules would require natural gas (and coal) power plants to reduce CO_2_ emissions drastically or retire. The principal technology by which those reductions are expected to be achieved is known as carbon capture and storage (CCS). While this technology has been endorsed by the United Nations’ Intergovernmental Panel on Climate Change, strong policy drivers failed to materialize, leaving it costly to build. To that end, Congress created the Section 45Q tax credit in 2008, increasing the credit amount in 2018, and again recently in the Inflation Reduction Act (IRA). Our research suggests that the use of CCS for some existing coal power plants to remove ≥90% of CO_2_ emissions is now economically viable.^15,16^

However, while the IRA updated the Section 45Q program to reflect contemporary market realities for coal, it neglected to recognize that, since 2008, the nation’s coal-fired generating capacity has been largely supplanted by natural gas power plants. For the Section 45Q credits to be effective going forward, additional modifications to the program are necessary.

In particular, the tax credits should be modified to ensure that the incentives provided to natural gas are economically equivalent to those provided to coal power plants.^17^ Because CCS costs are similar for both coal and natural gas power plants, but natural gas plants emit less CO_2_, the per-ton credit value they receive should be increased to provide comparable revenues. For the addition of CCS to a power plant to be viable, investors must also have a reasonable expectation of receiving the credits for their full 12-year term. With the aging coal fleet facing retirement, many of the otherwise-eligible coal plants may not continue to operate for that full term. In contrast, the average remaining life of the U.S. natural gas power plant fleet makes it ideally positioned to benefit from the program.^17^

If the underlying power plants face a significant risk of forced retirement before that point, however, the resulting uncertainty becomes toxic to investors. This regulatory uncertainty is a real risk for capital-intensive projects such as CCS.^18^ The prospect of a shifting regulatory landscape—and the virtually inevitable ensuing litigation—is likely to push owners simply to retire their plants rather than bear the risks.^19^ We emphasize that it is not sufficient merely to incentivize new carbon-removing technologies; the government must mitigate the uncertainty that the underlying generators will face until the full amount of the tax credits is monetized.

If maintaining CCS-abated natural gas capacity becomes impossible because those plants are pushed out of the market, only extremely costly or unreliable alternatives for electricity generation remain. There is also an additional benefit to maintaining natural gas power plants in the market. The Section 45Q credits can be leveraged now to exploit existing natural gas infrastructure to support CO_2_ hubs and build the foundations of a future hydrogen economy.^11,20^ The only alternatives are to build more nuclear plants, to require a massive expansion of costly battery storage or politically difficult transmission infrastructure, or to accept a less-reliable, less-resilient electric grid. Acting in haste now with dated and ill-designed policies will only drive out the very infrastructure the United States needs for a clean and reliable future.
